# Ecological implications of row width and cultivar selection on rice (*Oryza sativa*) and barnyardgrass (*Echinochloa crus-galli*)

**DOI:** 10.1038/s41598-024-76849-1

**Published:** 2024-10-22

**Authors:** Noah H. Reed, Thomas R. Butts, Jason K. Norsworthy, Jarrod T. Hardke, L. Tom Barber, Jason A. Bond, Hunter D. Bowman, Nick R. Bateman, Aurelie M. Poncet, Koffi B. J. Kouame

**Affiliations:** 1 Department of Crop, Soil, and Environmental Sciences, University of Arkansas System Division of Agriculture, Fayetteville, AR USA; 2 Department of Botany and Plant Pathology, Purdue University, West Lafayette, IN USA; 3 Department of Crop Soil and Environmental Sciences, University of Arkansas System Division of Agriculture, Stuttgart, AR USA; 4 Present Address: Department of Crop Soil and Environmental Sciences, University of Arkansas System Division of Agriculture, Lonoke, AR USA; 5 Department of Plant and Soil Sciences, Mississippi State University, Delta Research and Extension Center, Stoneville, MS USA; 6 Department of Entomology and Plant Pathology, Stuttgart, AR USA

**Keywords:** Canopy coverage, Density, Remote sensing, Seedbank, Weed seed production, Agroecology, Plant development, Plant ecology, Plant reproduction

## Abstract

Rice (*Oryza sativa* L.) producers in the Mid-south are experiencing difficulties with herbicide-resistant weeds such as barnyardgrass [*Echinochloa crus-galli* (L.) P. Beauv.]. As a result, methods that can alter *E. crus-galli* ecology are needed. This research evaluated the ecological implications of rice cultivar and row widths on crop and *E. crus-galli* growth. Overall, for *E. crus-galli*, as the row width increased, greater density, panicle counts, and seed production occurred. *Echinochloa crus-galli* density was 120% greater in a 38-cm row width than the 13-cm row width at the preflood rice stage. Reduced early-season rice canopy coverage in the wider row widths allowed for increased *E. crus-galli* densities. At the preharvest stage, *E. crus-galli* panicle counts were similar for the 13- and 19-cm rows. Row width did not affect rice yield, indicating wider row widths could be feasible agronomically, but additional weed management efforts would be needed because greater ecological advantages were obtained in narrower rows. Less *E. crus-galli* seed production occurred in competition with hybrid cultivars compared to inbred cultivars. Overall, the standard row width (19-cm) and hybrid cultivars would provide the greatest ecological advantage over *E. crus-galli*.

## Introduction

Globally, rice (*Oryza sativa* L.) production and consumption have increased significantly in the 2022/23 growing season compared to previous years, highlighting the need for more exportation of the crop from the United States (U.S.)^1^. Additionally, for the 2022/2023 season, Arkansas was the lead-producing state in the U.S., accounting for over 50% of rice production^2^. Weeds are major pests of rice production, causing yield reductions up to 96% when no management strategies are implemented^3^. In the late 1940s, farmers were introduced to a new weed control method, which consisted of chemical herbicides. The continuous use of these herbicides and the decrease in new chemistry led to herbicide-resistant weeds and the need for diversified approaches^4^.

Integrated pest management was first defined in 1967, although its concepts had been occurring for centuries prior, detailing the use of biological, physical, and selective chemical methods in managing certain pests^5^. Liebman and Gallandt (1997) coined the term “many little hammers” for use in integrated weed management (IWM), highlighting the importance of using several different management strategies instead of “one large hammer” or one method like herbicides to manage weeds more efficiently^6^. Herbicide-resistant weed populations and increasing regulations have renewed importance in IWM methods like using more competitive crop cultivars and manipulating row width to enhance crop ecology^7,8^. The most problematic weed in flooded and furrow-irrigated rice in Arkansas is barnyardgrass [*Echinochloa crus-galli* (L.) P. Beauv.]^9^. Due to herbicide resistance in *E. crus-galli*, other IWM options must be used to enhance the competitiveness of rice with this troublesome weed^10^.

Rice planted in Arkansas is drilled at a width ranging from 10 to 25 cm depending on the producers’ equipment and field conditions, but as the row width narrows, canopy closure occurs faster resulting in higher grain yield^11^. Previous research in other crops, such as soybean [*Glycine max* (L.) Merr.], soft red winter wheat (*Triticum aestivum* L.), and peanut (*Arachis hypogaea* L.) demonstrated that as row width narrows, crops more effectively compete with and have a greater ecological impact on weeds compared to wider row widths^12–14^. In soybean, a narrower row was more profitable than a wide row system because the crop canopied quicker over the soil and produced a greater yield^15,16^. Soybean yield increased by 10, 18, and 20% for 76, 51, and 25-cm rows, respectively, compared to 102-cm rows in field studies conducted in Illinois^17^. In a rice production system, the same results could be observed as other major crops grown in the U.S.

Little research has been conducted investigating weed competition from varying row widths in a drill-seeded rice production system. Dass et al. (2017) observed that rice planted in narrow row widths of 15 to 25 cm was more productive with reduced weed infestations; however, these narrow row widths were not able to suppress weeds completely but did allow for reduced herbicide use by up to 50% in some conditions^18^. In other research, a 8.2-cm row width increased rice yield compared to 19- and 38-cm widths^19^. A narrow width of 20-cm reduced weed density and seed production of *E. crus-galli* and junglerice (*Echinochloa colona* L.), which could be an important management tactic in reducing the soil seedbank, in return, reducing the number of herbicide-resistant weeds^20,21^. Additionally, a narrow row width in conjunction with a faster-growing rice cultivar could allow the crop to achieve canopy closure more rapidly, enhancing weed management in rice production^22^.

A soybean study conducted in Illinois found that different cultivars had variable responses in yield with early maturing cultivars in 17-cm rows producing the greatest yield^23^. One fundamental non-chemical control option for weeds is crop interference; the earlier the interference with the weed, the quicker it can prevent resource consumption from the pest^24^. Crop competition can be enhanced by using rice cultivars that have advantages of acquiring resources such as water, light, and space with higher tillering capabilities. This and other non-chemical methods such as narrow row width are being utilized in Australia for IWM^25^. Furthermore, non-chemical options such as narrow row widths and cultivar selection are being used in cotton to enhance competitiveness with weeds^26^.

Hybrid and inbred rice are both utilized in the Mid-South and Arkansas, with hybrid rice accounting for approximately 60% of production in the state; most of the rice in Arkansas is long-grain (91.7%), and the rest harvested is medium-grain (8.3%).^27^ There is evidence that certain rice cultivars have a greater probability of suppressing weeds and reducing herbicide use than other cultivars^28^. Hybrid rice cultivars have produced grain yields of 17 to 20% greater than inbred cultivars and could be more competitive against weeds to improve management^29,30^. Hybrid rice is also planted at lower seeding rates, with reduced nitrogen fertilizer, and more disease tolerance than inbred cultivars because of higher tillering capacity and faster growth characteristics allowing for lower input costs^22,31^. Additionally, yield losses caused by weed competition can range from 44 to 96% depending on conditions and the cultivar of rice grown^32^.

Due to the aforementioned weed management challenges in rice production, there is an established need for more diversified, ecologically-based management tactics. The objective of this research was to evaluate the impact of rice cultivar and row width on *E. crus-galli* density, panicle count, and seed production, rice canopy coverage, and yield.

## Materials and methods

### Field sites

Field experiments were conducted across four locations in 2021 and 2022 for a total of seven site-years. Sites consisted of the University of Arkansas Pine Bluff Small Farm Outreach Center near Lonoke, Arkansas (34.85°N, 91.88°W), the University of Arkansas System Division of Agriculture Pine Tree Research Station near Colt, Arkansas (35.13°N, 90.96°W), the Delta Research and Extension Center, Stoneville, MS (33.40°N, 90.86°W) in 2021 and 2022, and the University of Arkansas System Division of Agriculture Rohwer Research Station near Watson, Arkansas (33.79°N, 91.29°W) in 2022 only.

The soil at the Lonoke site was an Immanuel silt loam (fine-silty, thermic Oxyaquic Glossaqualfs) consisting of 14% sand, 72% silt, 14% clay, and 1.25% organic matter with a pH of 5.6. The soil at the Pine Tree site was a Calhoun silt loam (fine-silty, thermic Typic Glossaqualfs) consisting of 12% sand, 70% silt, 18% clay, and 1.02% organic matter with a pH of 5.6. The soil at the Stoneville site was a Sharkey clay (very-fine, thermic Chromic Epiaquerts) consisting of 2% sand, 32% silt, 66% clay, and 2.4% organic matter with a pH of 7.5. The soil at the Rohwer site was a Sharkey clay (very-fine, thermic Chromic Epiaquerts) consisting of 2% sand, 45% silt, 53% clay, and 1.98% organic matter with a pH of 6.8. The sites were drill-seeded with rice on the following dates: Lonoke, June 16, 2021, and May 16, 2022; Pine Tree, July 7, 2021, and June 7, 2022; Stoneville, May 26, 2021, and May 11, 2022; and Rohwer June 28, 2022.

Weed species at each field site were naturally-occurring populations present at the time the research was conducted. Each species, in particular *E. crus galli*, were identified through historical reference of the site and by the expert weed science authors of the manuscript. No voucher specimen was collected. Additionally, no permission was necessary to collect plant specimens as the research was conducted on university-owned research stations and *E. crus-galli* is classified as a noxious weed by the Arkansas Department of Agriculture^33^.

## Experimental design

The experiments were arranged as a randomized complete block split-plot design (16 treatments) replicated four times. Each experiment consisted of a whole plot factor of four row widths: 13-, 19-, 25-, and 38-cm. These row width treatments were selected as 19- and 25-cm row widths are currently commercially-available, a reduced width of 13-cm was hypothesized to aid in cultural weed management, and a 38-cm row width may become commercially-available in the future with enhancements in precision planting technology and may aid in facilitating crop rotation capabilities with reduced equipment inputs. The current industry standard for grain drills across the U.S. is a 19-cm row width. The subplot factor consisted of four rice cultivars: imidazolinone-resistant medium-grain inbred (CLM04, Horizon Ag LLC, Memphis, TN 38125), imidazolinone-resistant long-grain inbred (CLL16, Horizon Ag LLC, Memphis, TN 38125), conventional long-grain hybrid (RT7301, RiceTec Inc., Alvin, TX 77512), and imidazolinone-resistant long-grain hybrid (RT7521 FP, RiceTec Inc., Alvin, TX 77512). These cultivars were selected based upon commercial popularity in the state of Arkansas.

According to university recommendations, the inbred and hybrid rice cultivars were drill-seeded at different rates of 385 seeds m^− 2^ and 128 seeds m^− 2^, respectively^11^. The plots were 1.5 m wide and 7.6 m in length. The University of Arkansas standard recommendations were also used for nutrients, pests, and irrigation/flooding^11^.

High levels of weed infestation and previous survey results indicated commercial rice fields in Arkansas typically receive three to four herbicide applications including a preemergence and two to three postemergence applications^9^. As a result, the decision was made to apply a non-commercial herbicide program within this research targeting grass, sedge, and broadleaf weed species specific to each respective site-year. This non-commercial program included two herbicide applications (a preemergence and one postemergence) to allow assessment of the cultural factors but provide the opportunity for trials to be harvested for yield assessment. The herbicide applications were made by a CO_2_-pressurized sprayer using a tractor-mounted three-point sprayer or an All-Terrain Vehicle (ATV) equipped with AI 110015 nozzles (TeeJet Technologies, Glendale Heights, IL 60139) to deliver 94 L ha^− 1^ at 8 km hr^− 1^ at the Arkansas locations. At the Mississippi location, applications were made by a CO_2_-pressurized backpack sprayer calibrated to deliver 140 L ha^− 1^ at 5.6 km hr^− 1^ with TDXL 11002 nozzles (Greenleaf Technologies, Covington, LA 70434).

Across site-years, a preemergence application of clomazone at 315 g a.i. ha^− 1^ (Command^®^ 3ME, FMC Corporation, Philadelphia, PA 19104) and saflufenacil at 75 g a.i. ha^− 1^ (Sharpen^®^, BASF Corporation, Morrisville, NC 27709) was applied. Different postemergence applications were made across site-years depending on the weed species and density present in the experiment. The additional applications at the Lonoke and Pine Tree locations in 2021 consisted of cyhalofop at 313 g a.i. ha^− 1^ (Clincher^®^ SF, Corteva Agriscience LLC, Indianapolis, IN 46268) and halosulfuron + thifensulfuron at 35 + 4.5 g a.i. ha^− 1^ (Permit Plus^®^, Gowan, Yuma, AZ 85366). The postemergence application at the Lonoke site in 2022 was bentazon applied at 560 g a.i. ha^− 1^ (Basagran^®^, BASF Canada Inc., Mississauga, Ontario). The postemergence application at the Stoneville site in 2021 and 2022 was quinclorac at 420 g a.i. ha^− 1^ (Facet^®^, BASF Corporation, Morrisville, NC 27709). Applications at the Pine Tree and Rohwer locations in 2022 consisted of fenoxaprop at 122 g a.i. ha^− 1^ (Ricestar^®^ HT, Gowan, Yuma, AZ 85366), bispyribac-sodium at 3.5 g a.i. ha^− 1^ (Regiment^®^, Valent U.S.A., Walnut Creek, CA 94596), and halosulfuron + thifensulfuron at 35 + 4.5 g a.i. ha^− 1^ (Permit Plus^®^, Gowan, Yuma, AZ 85366).

## Data collection

*Echinochloa crus-galli* density was assessed from two 0.25 m^2^ quadrants per plot at the 5- to 6-leaf rice stage (preflood) and the preharvest stage. Density data were converted to a square meter scale for a better understanding of densities. A small, unmanned aircraft system (sUAS) [Inspire 2 (DJI Technology Co., LTD., Nanshan, Shenzhen, China)] was manually flown to take digital images overhead each plot at the preflood and panicle differentiation rice stages at the Lonoke and Pine Tree locations in 2022 only to assess canopy coverage. In 2021, technological complications caused the images not to be taken accurately to assess canopy coverage. At the Rohwer location in 2022, there was very high weed pressure, and the software could not discern the crop from weeds to evaluate the images accurately. At Stoneville, MS, technological difficulties did not allow for proper data collection. The images were captured at a height of 46 m across all plots for consistency in the analysis software. Aerial images were analyzed using FieldAnalyzer software (Green Research Services, LLC., Fayetteville, AR). Green pixel counts were measured in each plot to determine the canopy coverage percentage. Green pixel hue and saturation for the weeds and crops were different so the software was able to differentiate them in each plot. Before rice harvest, *E. crus-galli* panicles were harvested from two 0.25 m^2^ quadrants per plot, placed in paper bags, and subsequently averaged. *Echinochloa crus-galli* inflorescences were dried at 66 C for 3 to 5 d to constant mass. The panicles were then hand-threshed and cleaned to gather the *E. crus-galli* seed. The mass of 100 *E. crus-galli* seeds was recorded and divided by the total mass of cleaned seeds to determine the seed production per 0.25 m^2^ of each plot. Seed production was then converted to a m^2^ scale for ease of presentation. Rough rice grain yield was collected at harvest with a small-plot research combine. The entire width of the plot was harvested at the Lonoke, Rohwer, and Stoneville locations. At the Pine Tree location, two different plot combines were used according to the row width of the plot. A 51-cm header was used to harvest 2 rows of the 25-cm row width and 4 rows of the 13-cm width per plot. A 72-cm header harvested 2 rows of the 38-cm row width and 4 rows of the 19-cm width per plot.

## Statistical analyses

All data were subjected to analysis of variance (ANOVA). Site-year and block nested within site-year were run as random effects across all analyses to generate broader conclusions across the study. Row width and rice cultivar were considered fixed effects. *Echinochloa crus-galli* seed production and rough rice grain yield data were analyzed using the GLIMMIX procedure in JMP Pro 17.0 (SAS Institute Inc, Cary, NC) with a gaussian distribution. *Echinochloa crus-galli* density at preflood and preharvest panicle counts were analyzed using the GLIMMIX procedure with the Poisson distribution in JMP Pro 17.0. Rice canopy coverage was analyzed using SAS v9.5 (SAS Institute Inc, Cary, NC) with the GLIMMIX procedure and a beta distribution. All means were separated using Tukey’s honestly significant difference with an alpha value of 0.10.

## Results and discussion

Across all response variables, no interaction between row width and choice of cultivar was observed (Table 1). Across site-years at the preflood stage, row width affected *E. crus-galli* plant density. The lowest density at this stage was in the 13-cm row width with 15 plants m^− 2^ followed by 19 plants m^− 2^ in the 19-cm row width (Table 2). The 25- and 38-cm row widths had greater *E. crus-galli* densities compared to the 13- and 19-cm row widths but had similar results to each other with 28 and 33 plants m^− 2^, respectively (Table 2). *Echinochloa crus-galli* density was 120% greater in the 38-cm row width compared to the 13-cm row width. Hence, to delay and reduce the emergence of *E. crus-galli*, rice in a narrower row width could possess the ability to outcompete the weed like findings elsewhere^20^.

**Table 1:** Analysis of variance results for *Echinochloa crus-galli* density at preflood and *E. crus-galli* panicle counts at preharvest, rice canopy coverage at preflood and panicle differentiation, *E. crus-galli* seed production before harvest, and rough rice yield across site-years.^a, b^

	*E. crus-galli* density	*E. crus-galli* panicle count	Rice canopy coverage			
Source	Preflood	Preharvest	Preflood	Panicle differentiation	*E. crus-galli* seed	Rough rice yield
	*P > F*					
Row	**< 0.0001**	**< 0.0001**	**0.0007**	**0.0043**	**0.0279**	0.4866
Cultivar	0.3058	**< 0.0001**	**0.0093**	**0.0560**	**0.0051**	**< 0.0001**
Row*Cultivar	0.3425	0.6204	0.8874	0.8060	0.9586	0.9976

^a^Bolded values indicate statistical significance at α = 0.10

^b^Rice canopy coverage is from the 2022 Lonoke and Pine Tree site-years only due to excessive weed pressure and software limitations at other site-years. *Echinochloa crus-galli* density, panicle counts, and seed production is from 5 site-years.

**Table 2:** *Echinochloa crus-galli* density at preflood, panicle count at preharvest, and seed production across five site-years^a^.

Main effect	*E. crus-galli* density	*E. crus-galli *panicle count	*E. crus-galli *seed production (# m^-2^)
	Preflood (# m^-2^)	Preharvest (# m^-2^)	
Row width (cm)			
13	15 c	11 c	23,300 b
19	19 b	12 bc	21,130 b
25	28 a	14 b	24,600 ab
38	33 a	19 a	30,960 a
Cultivar			
CLM04	27 a	16 a	29,520 a
CLL16	25 a	17 a	30,960 a
RT7301	25 a	11 b	19,670 b
RT7521 FP	24 a	11 b	20,990 b

^a^Means followed by the same letter within a main effect and column are not different based on Tukey’s HSD (α=0.10).

While there were no differences in *E. crus-galli* density across cultivars at the preflood stage, some conclusions can still be drawn. No cultivar effect at this rice stage may be because it was still too early in the life cycle of rice to see competitive differences. It has been suggested that hybrid rice has a higher tillering capacity compared to an inbred cultivar^34^. At this point in the life cycle (only 4 to 5 leaves), there was not enough enhanced growth of hybrid rice compared to inbred to see impacts on *E. crus-galli* density.

At the preharvest rice stage, similar trends across row width were observed for the *E. crus-galli* panicle count assessment as the preflood plant density. Generally, as row width increased, *E. crus-galli* panicles also increased. The 13- and 19-cm row widths had the fewest panicle counts (11 and 12 panicles m^− 2^, respectively) (Table 2). An increase of panicles was present in the wider row widths where the 25-cm width had 14 panicles m^− 2^ and the 38-cm width had 19 panicles m^− 2^. These results were similar to previous research conducted in soybean, where in wider rows, more solar radiation penetrated to the soil surface, allowing weeds to grow and compete with the crop^35^. At the preharvest stage, cultivar selection influenced *E. crus-galli* panicle counts. There were more *E. crus-galli* panicles (16 and 17 panicles m^− 2^) in the inbred cultivars of CLM04 and CLL16, respectively, than the hybrid cultivars RT7301 and RT7521 FP (11 and 11 panicles m^− 2^, respectively) (Table 2). The high vigor, drought tolerance, greater tillering capacity, and higher disease resistance could help explain how the hybrid cultivars could better compete with *E. crus-galli.*

One of the best management practices in preventing herbicide resistance is reducing the soil seedbank, which in turn reduces the spread of resistant genes^36^. Both row width and cultivar selection affected *E. crus-galli* seed production (Table 1). The lowest amount of *E. crus-galli* seed produced (21,130 seeds m^− 2^) occurred in the 19-cm row width but was not different from the 13-cm (23,300 seeds m^− 2^) and 25-cm (24,600 seeds m^− 2^) widths (Table 2). There was increased *E. crus-galli* seed production (30,960 seeds m^− 2^) in the wider width of 38-cm compared to the narrower row widths, illustrating that the widest width led to increased escapes and allowed more seed to be returned to the soil seedbank. As a result, 13-cm through 25-cm row widths could be utilized to reduce weed seeds return to the soil seedbank. The 38-cm row width would likely enhance the risk of increasing the soil seedbank, potentially leading to increased herbicide resistance concerns the following crop year without implementing additional weed management measures. A study in soybean found similar results of increased weed seed production as the crop row width increased^12^. Seed production from *E. crus-galli* was similarly affected by cultivar selection as the preharvest panicle counts. The greatest number of *E. crus-galli* seeds produced were in the CLM04 and CLL16 inbred cultivars with 29,520 and 30,960 seeds m^− 2^, respectively. Hybrid cultivars (RT7301 and RT7521 FP) resulted in the fewest amount of *E. crus-galli* seeds with 19,670 and 20,990 seeds m^− 2^, respectively (Table 2). As a result, this present research highlights the ecological advantages of hybrid rice cultivars to enhance competitiveness with weeds in addition to their documented yield increases compared with inbred cultivars^34^.

**Table 3:** Rice canopy coverage at preflood and panicle differentiation across the Lonoke and Pine Tree 2022 site-years; rough rice yield across all seven site-years^a^.

Main effect	Rice canopy coverage		Rough rice yield kg ha^− 1^
	Preflood %	Panicle differentiation %	
Row width (cm)			
13	39 a	79 a	9,650 a
19	32 ab	77 a	9,970 a
25	31 b	73 ab	9,850 a
38	26 b	67 b	9,110 a
Cultivar			
CLM04	26 b	74 ab	7,880 c
CLL16	32 ab	70 b	8,960 bc
RT7301	34 a	72 ab	10,110 ab
RT7521 FP	36 a	79 a	11,630 a

^a^Means followed by the same letter within a main effect and column are not different based on Tukey’s HSD (α = 0.10).

During the preflood rice stage, both row width and cultivar selection affected rice canopy coverage (Table 1). The greatest canopy coverage (39%) was observed from the 13-cm row width (Table 3). As row width increased, canopy coverage percentage decreased. The 38-cm row width had the lowest canopy coverage percentage (26%). Even this early in the lifecycle of rice, the wider widths were not able to reach an equal canopy closure as narrower widths, allowing for more weeds to germinate and grow as evidenced by the increased E. crus-galli densities also observed at this timing (Table 2). The greatest canopy coverage from cultivar selection at the preflood timing was observed from the RT7521 FP hybrid cultivar (36%) (Table 3). The other long-grain cultivars (RT7301 34% and CLL16 32%) had a slight numerical decrease in canopy coverage but were not statistically different from RT7521 FP. However, the inbred medium-grain (CLM04) canopy coverage (26%) was 10 percentage points less than RT7521 FP at the preflood rice stage. These results provided insight that before the rice was taken to flood, hybrid rice enhanced growth to produce a greater canopy coverage compared to the medium-grain inbred cultivar. Rice with greater canopy coverage also has increased water use efficiency and resulted in increased yields.^37^ Additionally, this early season enhancement of canopy coverage may help explain the reduced *E. crus-galli* panicle counts and seed production from hybrid cultivars following season-long competition despite not impacting density at the preflood stage (Table 2).

The panicle differentiation rice stage is classified as the last vegetative stage and the start of the reproductive stage where vegetative production ceases and the seed head begins formation^38^. During this timing, differences in rice canopy coverage were observed for both row width and cultivar (Table 1). The greatest canopy coverage was observed from the narrowest row width (13 cm) at 79% (Table 3). The least canopy coverage (67%) was observed from the 38-cm width. The 13- through 25-cm widths produced equivalent canopy coverage with an average of 76%. Similar results were found in a study of corn (*Zea mays* L.) where the row width of 38 cm reduced light transmittance to the soil surface one week earlier than the 76-cm width^39^. Stopping light transmittance to the soil and minimizing diurnal temperature fluctuations with canopy closure could explain why lower *E. crus-galli* densities were observed in narrower row widths (Table 2)^40,41^. For cultivar selection impacts, the greatest canopy coverage occurred with RT7521 FP (79%) followed by CLM04 (74%), RT7301 (72%), and CLL16 (70%), respectively (Table 3). While RT7521 FP had the greatest canopy coverage, all cultivars were at or above 70%. Hence, some cultivars may be slower in canopy development (CLM04), but likely can obtain equivalent canopy coverage percentages by the panicle differentiation growth stage in which most of the vegetative growth has concluded. However, without implementing additional weed management strategies, delayed canopy development likely helps to explain the increase in panicle counts and seed production over the course of a growing season (Table 2).

Rough rice yield was collected across all site-years. No difference occurred between row widths, but there was a cultivar effect (Table 1). Across site-years and averaged across row width, the long-grain hybrids RT7521 FP (11,630 kg ha^− 1^) and RT7301 (10,110 kg ha^− 1^) produced the greatest yield (Table 3). In contrast, the inbred medium-grain cultivar, CLM04 (7,880 kg ha^− 1^), had the lowest yield. Overall, hybrid rice cultivars generally produced greater yields than inbred counterparts, likely due to increased tolerance to stress and drought resistance, as well as increased early-season canopy coverage (Table 3). Since there were no row width impacts on yield (all yields between 9,110 and 9,980 kg ha^− 1^, Table 3) it would indicate that wider row widths may be feasible agronomically. This was previously observed in soybean where row width did not affect crop yield^12^. However, weed management would need to be considered as wider row widths will require additional efforts to reduce weed seed from returning to the soil seed bank affecting long-term weed management (Table 2).

## Conclusions

Overall, results indicated that as rice row width increased, *E. crus-galli* density, panicle counts, and seed production increased. However, it should be acknowledged that the delayed planting dates within this research and resulting *E. crus-galli* emergence timings may have influenced the overall results. Future research should explore the role of rice planting date and *E. crus-galli* emergence timings paired with these cultural management efforts to further examine their ecological impacts. Although decreased *E. crus-galli* density preflood was observed in the 13-cm row width compared to the 19-cm row width (4 plants m^− 2^), there were no observed differences between these treatments in any other collected response variables. Further, the additional 9 plants m^− 2^ in the 25-cm row width compared with the 19-cm row width preflood would lead to increased selection pressure for postemergence herbicide resistance, and there are currently limited grain drills operated with either a 13- or 25-cm row width. Therefore, the 19-cm row width would be recommended as it is not economically viable to purchase new equipment on alternative row widths when similar ecological impacts and yields were obtained. It may be advisable to plant hybrid cultivars for quicker canopy formation and greater competitiveness with *E. crus-galli* than the inbred cultivars. However, future research should evaluate the economic considerations of this weed management benefit versus the increased seed costs. If a 38-cm precision planter does become commercially available in the future to aid in crop rotation efficiency, additional research is needed to identify a more targeted weed management program for the wider widths to reduce weed seed returns to the soil seedbank, evaluate the economics of seeding rates and greater management costs compared to standard narrower widths, and understand the long-term ecological weed influence of crop rotation with a rice production system.

## Data Availability

The datasets generated and analyzed during the current study are available from the corresponding author on reasonable request.
